# Prevalence and clinical significance of Claudin-3 expression in cancer: a tissue microarray study on 14,966 tumor samples

**DOI:** 10.1186/s40364-024-00702-w

**Published:** 2024-12-10

**Authors:** Seyma Büyücek, Nina Schraps, Anne Menz, Florian Lutz, Viktoria Chirico, Florian Viehweger, David Dum, Ria Schlichter, Andrea Hinsch, Christoph Fraune, Christian Bernreuther, Martina Kluth, Claudia Hube-Magg, Katharina Möller, Viktor Reiswich, Andreas M. Luebke, Patrick Lebok, Sören Weidemann, Guido Sauter, Maximilian Lennartz, Frank Jacobsen, Till S. Clauditz, Andreas H. Marx, Ronald Simon, Stefan Steurer, Eike Burandt, Natalia Gorbokon, Sarah Minner, Till Krech, Morton Freytag

**Affiliations:** 1 Institute of Pathology, University Medical Center Hamburg-Eppendorf, Martinistr. 52, Hamburg, 20246 Germany; 2 General, Visceral and Thoracic Surgery Department and Clinic, University Medical Center Hamburg-Eppendorf, Hamburg, Germany; 3 Institute of Pathology, Clinical Center Osnabrueck, Osnabrueck, Germany; 4 Department of Pathology, Academic Hospital Fuerth, Fuerth, Germany

**Keywords:** CLDN3, Tissue microarray, Cancer, Renal cell carcinoma, Biomarker

## Abstract

**Background:**

Claudin-3 (CLDN3) participates in the formation of the tight-junctions (TJs) that regulate intercellular permeability. Altered CLDN3 expression has been linked to tumor progression in multiple tumor types. Despite its widespread expression in normal epithelial cells, CLDN3 is considered an attractive drug target candidate, since it may be more accessible in cancer cells than in normal cells due to their less orchestrated cell growth.

**Methods:**

To comprehensively determine the prevalence of CLDN3 expression in cancer, a tissue microarray containing 14,966 samples from 133 different tumor types and subtypes as well as 608 samples of 76 different normal tissue types was analyzed by immunohistochemistry.

**Results:**

CLDN3 immunostaining was observed in 8,479 (68.9%) of 12,314 analyzable tumors, including 11.6% with weak, 6.2% with moderate, and 51.1% with strong positivity. CLDN3 staining was found in 96 of 133 tumor categories, 80 of which contained at least one strongly positive case. CLDN3 positivity was most seen in neuroendocrine neoplasms (92–100%) and in adenocarcinomas (67–100%), tumors of the female genital tract, including various subtypes of ovarian and endometrial carcinoma (up to 100%), as well as different subtypes of breast cancer (95.3–100%). CLDN3 positivity was less common in squamous cell carcinomas (0–43.2%) and mainly absent in melanoma, mesenchymal, and hematolymphatic neoplasms. In clear cell renal cell carcinoma (ccRCC), low CLDN3 was strongly linked to poor ISUP (*p* < 0.0001), Fuhrman (*p* < 0.0001), and Thoenes (*p* < 0.0001) grades, advanced pT category (p < 0.0001), high UICC stage (*p* = 0.0006) and distant metastasis (*p* = 0.0011), as well as shortened overall (*p* = 0.0118) and recurrence-free (*p* < 0.0001) survival. In papillary RCC (pRCC), low CLDN3 was associated with poor grade (*p* < 0.05), high pT (*p* = 0.0273) and distant metastasis (*p* = 0.0357). In urothelial carcinoma high CLDN3 was linked to high grade (*p* < 0.0001) and nodal metastasis (*p* = 0.0111). The level of CLDN3 staining was unrelated to parameters of tumor aggressiveness in pancreatic, gastric, and breast cancer.

**Conclusion:**

In conclusion, our data demonstrate significant levels of CLDN3 expression in many different tumor entities and identify reduced CLDN3 expression as a potential prognostic marker in RCC.

**Supplementary Information:**

The online version contains supplementary material available at 10.1186/s40364-024-00702-w.

## Introduction

Claudin-3 (CLDN3) is one of 27 known members of the claudin family [1]. Together with occludin and other junctional adhesion molecules, the claudins form the tight-junctions (TJs) that regulate intercellular permeability [2]. Claudins can be distinguished into paracellular barrier forming and pore forming claudins allowing for controlled diffusion of ions and water through TJs [3]. TJs display characteristic individual compositions and ratios of different claudins which define individual “penetrability properties” in different tissues and cell types [2, 4]. CLDN3 is a rather ubiquitously expressed barrier forming claudin which occurs in the intestine and many other epithelial tissues [5, 6].

Despite their widespread expression in normal cells, TJ components are considered attractive drug target candidates, since they may be more accessible in cancer cells than in normal cells. In normal epithelia, the accessibility of TJ proteins is limited by the orchestrated cell growth, the protection of individual TJ proteins by intact TJ structures, and the predominant expression of TJs at apical surfaces [7–10]. The misorientation of the cell division in cancerous tissues results in a markedly higher exposure of TJ components [7, 10, 11]. The expression of CLDN3 in cancer has been analyzed in more than 45 studies using immunohistochemistry (IHC). Aberrations of CLDN3 expression have been reported to occur in colorectal [12], breast [13–15], ovarian [16, 17], prostatic [18, 19], gastric [20–22], hepatic [23] and pulmonary cancers [24]. Several of these studies have found a link between either elevated [14, 18, 25] or reduced [19, 23, 24] CLDN3 expression levels and poor prognosis of cancer patients. It is of note that the reported rates of CLDN3 positivity varied considerably between studies. For example, the range of reported CLDN3 positive cases ranged from 25 to 73.6% in gastric cancer [20, 21], from 32 to 95% in breast cancer of no special type [26, 27], and from 41.4 to 97.0% in pulmonary adenocarcinoma [28, 29]. Such conflicting data between studies are typically caused by the use of different antibodies, IHC protocols, and criteria to define CLDN3 positivity.

To better understand the prevalence and potential clinical significance of CLDN3 expression in cancer, a comprehensive study analyzing a large number of neoplastic and non-neoplastic tissues under highly standardized conditions is needed. Therefore, CLDN3 expression was analyzed in more than 14,500 tumor tissue samples from 133 different tumor types and subtypes as well as 76 non-neoplastic tissue categories by IHC in a tissue microarray (TMA) format in this study.

## Material and methods

### Tissue Microarrays (TMAs)

The normal tissue TMA was composed of 8 samples from 8 different donors for each of 76 different normal tissue types (608 samples on one slide). The cancer TMAs contained a total of 14,966 primary tumors from 133 tumor types and subtypes. Detailed histopathological data on grade, pathological tumor stage (pT) or pathological lymph node status (pN) were available from breast cancers (*n* = 600), urothelial carcinomas (*n* = 829), ovarian cancers (*n* = 344), endometroid endometrial cancers (*n* = 182), thyroid (*n* = 518), gastric (*n* = 327), and pancreatic carcinomas (*n* = 598) as well as clear cell (*n* = 1,224) and papillary (*n* = 310) renal cell carcinomas (ccRCC, pRCC). Clinical follow up data were available from 789 patients with ccRCC and from 177 patients with pRCC with a median follow-up time of 48.0 and 50.5 months (range 1–250 and 1–247). The composition of both normal and cancer TMAs is described in detail in the results section. All samples were from the archives of the Institute of Pathology, University Medical Center Hamburg, Germany, the Institute of Patholgy, Clinical Center Osnabrueck, Germany, and Department of Pathology, Academic Hospital Fuerth, Germany. Tissues were fixed in 4% buffered formalin and then embedded in paraffin. The TMA manufacturing process was described earlier in detail [30, 31]. In brief, one tissue spot (diameter: 0.6 mm) per patient was used. The use of archived remnants of diagnostic tissues for TMA manufacturing, their analysis for research purposes, and the use of patient data were according to local laws (HmbKHG, §12) and analysis had been approved by the local ethics committee (Ethics commission Hamburg, WF-049/09). All work has been carried out in compliance with the Helsinki Declaration.

### Immunohistochemistry (IHC)

Freshly cut TMA sections were immunostained on one day and in one experiment. Slides were deparaffinized with xylol, rehydrated through a graded alcohol series and exposed to heat-induced antigen retrieval for 5 min in an autoclave at 121 °C in pH 7.8 Tris–EDTA-Citrat (TEC) puffer. Endogenous peroxidase activity was blocked with Dako REAL Peroxidase-Blocking Solution (Agilent Technologies, Santa Clara, CA, USA; #S2023) for 10 min. Primary antibody specific against CLDN3 protein (rabbit recombinant monoclonal, HMV-309, ardoci GmbH, Hamburg, Germany) was applied at 37 °C for 60 min at a dilution of 1:150. For the purpose of antibody validation, the normal tissue TMA was also analyzed by the rabbit recombinant monoclonal CLDN3 antibody EPR19971 (Abcam Limited, Cambridge, GB) at a dilution of 1:40 and an otherwise identical protocol. Bound antibody was then visualized using the Dako REAL EnVision Detection System Peroxidase/DAB + , Rabbit/Mouse kit (Agilent Technologies, Santa Clara, CA, USA; #K5007) according to the manufacturer’s directions. The sections were counterstained with hemalaun. IHC scoring was predefined and has been used in multiple previous studies [32–34]. For tumor tissues, the percentage of positive neoplastic cells was estimated, and the staining intensity was semi-quantitatively recorded (0, 1 + , 2 + , 3 +) [35]. For statistical analyses, the staining results were categorized into four groups. Tumors without any staining were considered negative. Tumors with 1 + staining intensity in ≤ 70% of tumor cells and 2 + intensity in ≤ 30% of tumor cells were considered weakly positive. Tumors with 1 + staining intensity in > 70% of tumor cells, 2 + intensity in 31–70%, or 3 + intensity in ≤ 30% of tumor cells were considered moderately positive. Tumors with 2 + intensity in > 70% or 3 + intensity in > 30% of tumor cells were considered strongly positive. The analysis by one pathologist enables the best possible consistency of interpretation within the study. A possible impact of interobserver variation was excluded as much as possible by a four-tier categorization of tumor staining. Although interobserver variation is common in TMA studies between 1 + and 2 + there is little discrepancies between 0 + and 3 + .

### Statistics

Statistical calculations were performed with JMP17® software (SAS®, Cary, NC, USA). Contingency tables and the chi^2^-test were performed to search for associations between CLDN3 immunostaining and tumor phenotype. Survival curves were calculated according to Kaplan–Meier. The Log-Rank test was applied to detect significant differences between groups.

## Results

### Technical issues

A total of 12,314 (82.3%) of 14,966 tumor samples were interpretable in our TMA analysis. Non-interpretable samples demonstrated lack of unequivocal tumor cells or lack of entire tissue spots. A sufficient number of samples (≥ 4) of each normal tissue type was evaluable.

### CLDN3 immunostaining in normal tissues

**Fig. 1:**
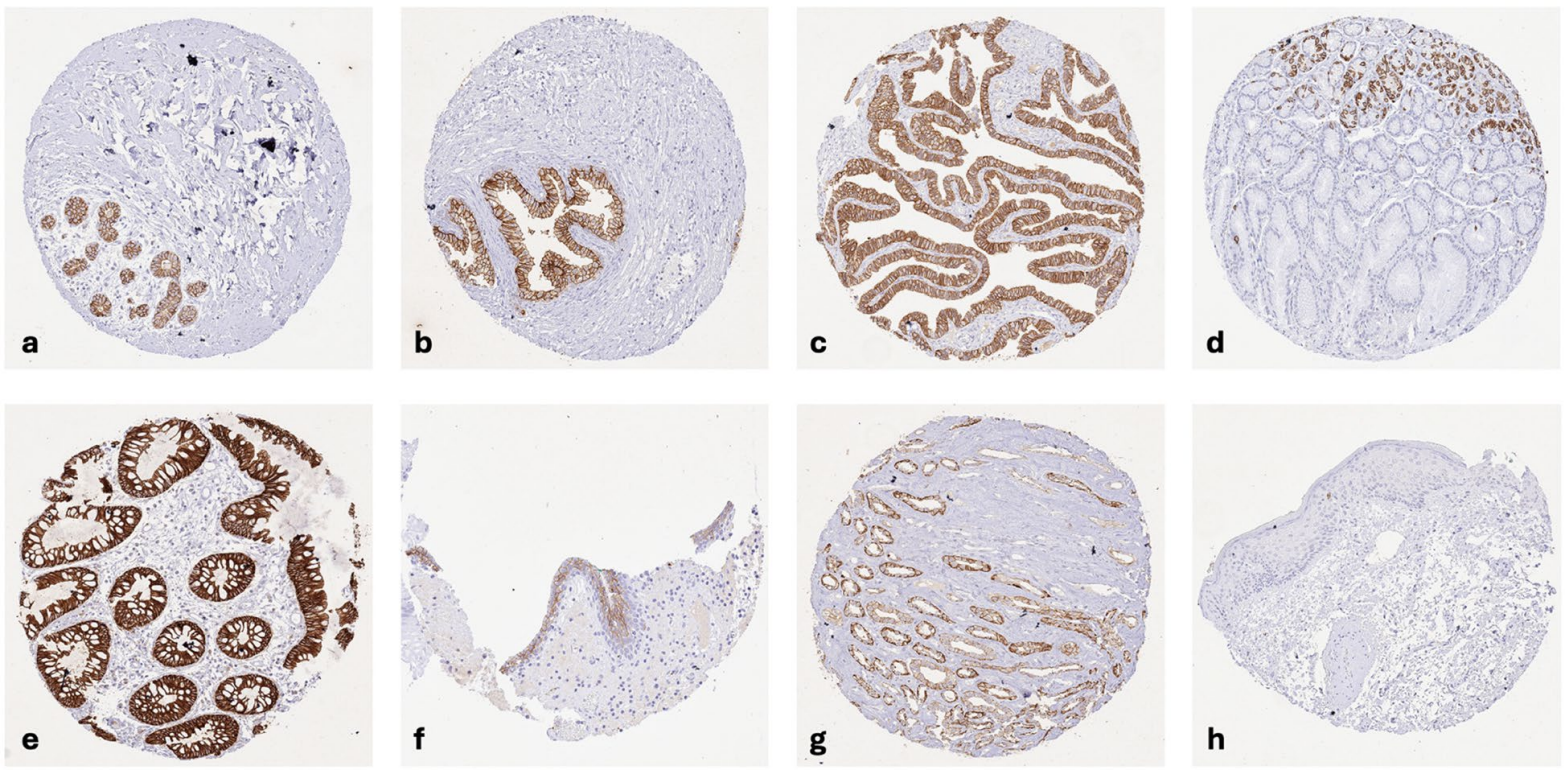
CLDN3 immunostaining of normal tissues. The panels show a strong membranous CLDN3 immunostaining of the luminal cells of breast glands (**a**) and of the prostate (**b**), epithelial cells of the fallopian tube (**c**), a small subset of gastric epithelial cells in the neck and in glandular pits (**d**), epithelial cells of the colorectum (**e**), the upper half of urothelial cells (**f**), and in collecting ducts of the kidney medulla (**g**) while CLDN3 staining is absent in squamous epithelial cells of the epidermis (**h**)

CLDN3 immunostaining was predominantly membranous. CLDN3 staining was particularly strong in luminal cells of breast glands, prostate, and seminal vesicle, follicular cells of the thyroid, respiratory epithelial cells, glandular cells of salivary glands, a small subset of gastric epithelial cells in the neck and in glandular pits, all epithelial cells of the small intestine and the colorectum, bile ducts in the liver and gallbladder epithelium, acinar cells of the pancreas, collecting ducts of the kidney, most epithelial cells in the cauda epididymis, epithelial cells of endometrium glands, the fallopian tube, and the endocervix (predominantly basolateral), megakaryocytes of the bone marrow, subsets of high endothelial venules and of monocytic cells in germinal centers of lymph nodes, as well as in squamous epithelial cells of tonsil crypts and corpuscles of Hassall’s in the thymus. A less intense, weak to moderate membranous CLDN3 staining was observed in the urothelium (predominantly in the upper half), epithelial cells of the parathyroidal gland, few epithelial cells of the adrenal gland, hepatocytes (predominantly at the bile secreting apical membrane), excretory ducts of salivary glands, islets cells of the pancreas, chief cells in the corpus epididymis, some renal tubular cells, hepatocytes, a large subset of corpus luteum cells of the ovary, pneumocytes, a subset of cells in the white pulp of the spleen, and the syncytiotrophoblast (surface membrane) of the first trimenon placenta. CLDN3 staining was absent in squamous epithelial cells of the epidermis, the ectocervix, and the esophagus, amnion, chorion, all muscle cells, and the brain. Representative images are shown in Fig. 1. All cell types identified as CLDN3 positive by HMV-309 were also positive by using EPR19971, although the signal was less intense for EPR19971 even at a dilution of 1:40 (Supplementary Fig. 1).

### CLDN3 immunostaining in neoplastic tissues

**Fig. 2:**
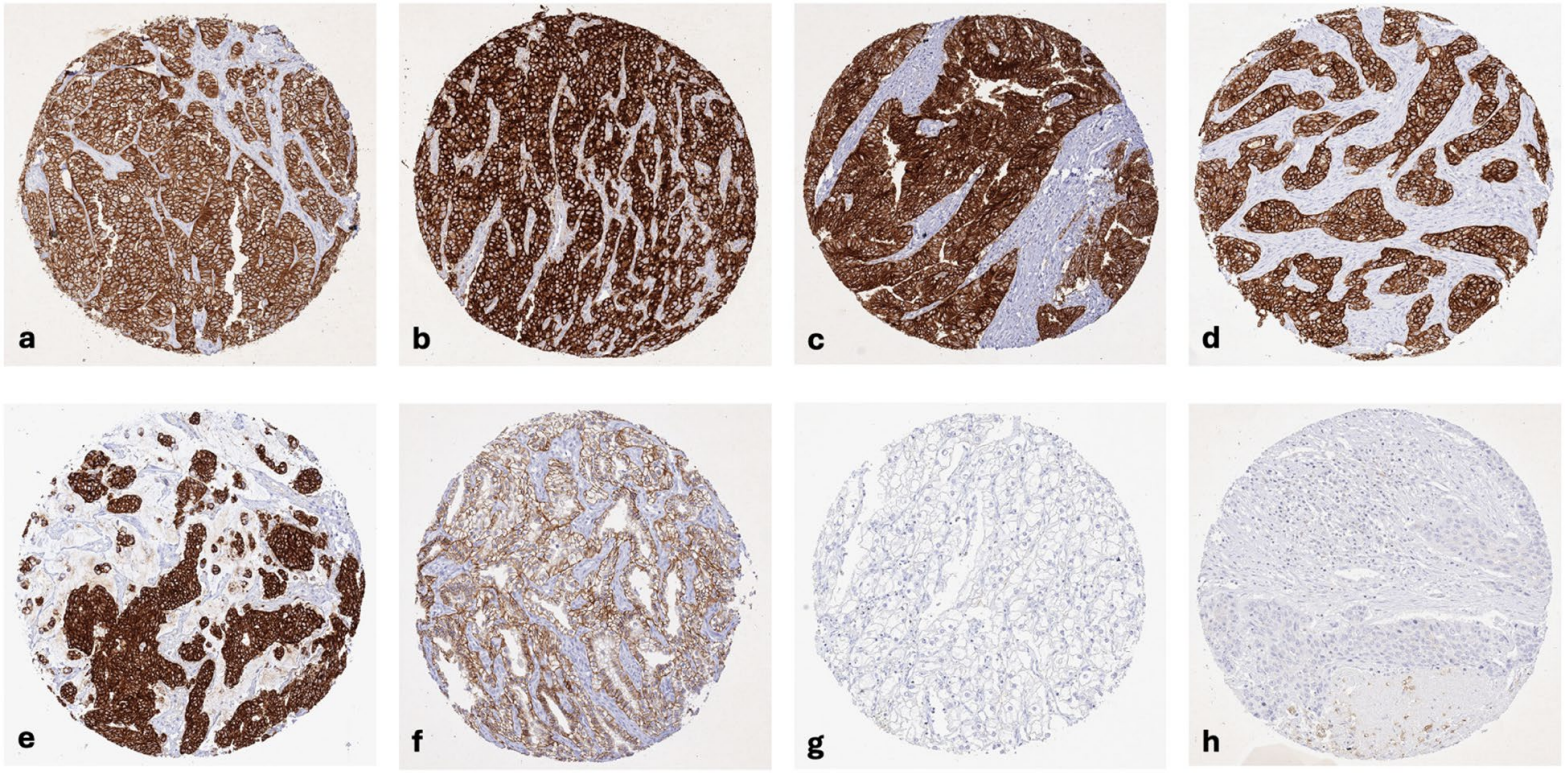
CLDN3 immunostaining in cancer. CLDN3 immunostaining was purely membranous in most tumors, with some showing an additional cytoplasmic positivity. The panels show a strong CLDN3 positivity in cancer cells of a neuroendocrine tumor of the appendix (**a**), an adenocarcinoma of the prostate (**b**), an endometrioid endometrial carcinoma (**c**), an invasive breast cancer of no special type (**d**), a muscle-invasive urothelial carcinoma (**e**), and clear cell renal cell carcinoma (**f**). CLDN3 staining is lacking in another clear cell renal cell carcinoma (**g**) and squamous cell carcinoma of the lung (**h**)

CLDN3 staining was observed in 8,479 (68.9%) of 12,314 analyzable tumors, including 11.6% with weak, 6.2% with moderate, and 51.1% with strong staining intensity. CLDN3 staining varied both in intensity and in its pattern between samples. Most CLDN3 positive tumors showed a purely membranous staining pattern but some tumors showed an additional cytoplasmic positivity. Representative images are shown in Fig. 2.

At least an occasional weak CLDN3 positivity was detected in 96 of 133 tumor categories and 80 categories included at least one case with strong CLDN3 positivity (Table 1).

**Table 1:** CLDN3 immunostaining in human tumors

			**CLDN3 immunostaining**				
	**Tumor entity**	on TMA (n)	analyzable (n)	negative (%)	weak (%)	moderate (%)	strong (%)
**Tumors of the skin**	Basal cell carcinoma of the skin	41	13	100.0	0.0	0.0	0.0
	Squamous cell carcinoma of the skin	95	80	93.8	6.3	0.0	0.0
	Malignant melanoma	19	19	100.0	0.0	0.0	0.0
	Malignant melanoma lymph node metastasis	86	82	98.8	1.2	0.0	0.0
	Merkel cell carcinoma	2	2	50.0	0.0	50.0	0.0
**Tumors of the head and neck**	Squamous cell carcinoma of the larynx	109	89	78.7	11.2	6.7	3.4
	Squamous cell carcinoma of the pharynx	60	41	65.9	24.4	0.0	9.8
	Oral squamous cell carcinoma (floor of the mouth)	130	102	84.3	4.9	4.9	5.9
	Pleomorphic adenoma of the parotid gland	50	45	37.8	31.1	20.0	11.1
	Warthin tumor of the parotid gland	49	48	2.1	31.3	43.8	22.9
	Basal cell adenoma of the salivary gland	15	15	20.0	13.3	20.0	46.7
**Tumors of the lung, pleura and thymus**	Adenocarcinoma of the lung	196	130	3.8	3.8	4.6	87.7
	Squamous cell carcinoma of the lung	80	45	71.1	17.8	2.2	8.9
	Mesothelioma, epithelioid	40	24	95.8	4.2	0.0	0.0
	Mesothelioma, biphasic	29	17	100.0	0.0	0.0	0.0
	Thymoma	29	29	69.0	6.9	13.8	10.3
	Lung, neuroendocrine tumor (NET)	29	25	8.0	0.0	8.0	84.0
**Tumors of the female genital tract**	Squamous cell carcinoma of the vagina	30	26	76.9	11.5	3.8	7.7
	Squamous cell carcinoma of the vulva	107	87	90.8	3.4	2.3	3.4
	Squamous cell carcinoma of the cervix	88	74	56.8	31.1	5.4	6.8
	Adenocarcinoma of the cervix	23	23	4.3	4.3	4.3	87.0
	Endometrioid endometrial carcinoma	288	261	0.4	5.7	10.3	83.5
	Endometrial serous carcinoma	36	32	0.0	0.0	6.3	93.8
	Carcinosarcoma of the uterus	57	46	21.7	17.4	4.3	56.5
	Endometrial carcinoma, high grade, G3	13	13	30.8	7.7	15.4	46.2
	Endometrial clear cell carcinoma	9	8	0.0	12.5	0.0	87.5
	Endometrioid carcinoma of the ovary	93	71	0.0	4.2	4.2	91.5
	Serous carcinoma of the ovary	530	445	0.2	1.3	2.7	95.7
	Mucinous carcinoma of the ovary	75	51	19.6	19.6	7.8	52.9
	Clear cell carcinoma of the ovary	51	40	0.0	5.0	0.0	95.0
	Carcinosarcoma of the ovary	47	36	19.4	11.1	5.6	63.9
	Granulosa cell tumor of the ovary	44	42	90.5	4.8	2.4	2.4
	Leydig cell tumor of the ovary	4	4	100.0	0.0	0.0	0.0
	Sertoli cell tumor of the ovary	1	1	0.0	100.0	0.0	0.0
	Sertoli Leydig cell tumor of the ovary	3	3	66.7	0.0	33.3	0.0
	Steroid cell tumor of the ovary	3	3	66.7	0.0	0.0	33.3
	Brenner tumor	32	26	88.5	7.7	0.0	3.8
**Tumors of the breast**	Invasive breast carcinoma of no special type	499	345	0.3	13.9	9.6	76.2
	Lobular carcinoma of the breast	150	107	4.7	17.8	16.8	60.7
	Medullary carcinoma of the breast	8	7	0.0	14.3	0.0	85.7
	Tubular carcinoma of the breast	2	1	0.0	0.0	100.0	0.0
	Mucinous carcinoma of the breast	7	4	0.0	0.0	0.0	100.0
**Tumors of the digestive system**	Adenomatous polyp, low-grade dysplasia	50	25	0.0	0.0	0.0	100.0
	Adenomatous polyp, high-grade dysplasia	50	38	0.0	0.0	0.0	100.0
	Adenocarcinoma of the colon	2483	2219	0.1	0.9	1.0	98.0
	Gastric adenocarcinoma, diffuse type	215	189	23.3	9.5	2.6	64.6
	Gastric adenocarcinoma, intestinal type	215	184	9.8	8.7	10.9	70.7
	Gastric adenocarcinoma, mixed type	62	50	8.0	14.0	8.0	70.0
	Adenocarcinoma of the esophagus	83	76	6.6	7.9	10.5	75.0
	Squamous cell carcinoma of the esophagus	76	69	68.1	17.4	4.3	10.1
	Squamous cell carcinoma of the anal canal	91	65	83.1	10.8	0.0	6.2
	Cholangiocarcinoma	58	46	15.2	21.7	23.9	39.1
	Gallbladder adenocarcinoma	51	44	6.8	20.5	11.4	61.4
	Gallbladder Klatskin tumor	42	27	22.2	33.3	25.9	18.5
	Hepatocellular carcinoma	312	274	11.7	56.6	14.6	17.2
	Ductal adenocarcinoma of the pancreas	659	415	33.0	33.3	12.5	21.2
	Pancreatic/Ampullary adenocarcinoma	98	65	12.3	7.7	10.8	69.2
	Acinar cell carcinoma of the pancreas	18	17	5.9	17.6	11.8	64.7
	Gastrointestinal stromal tumor (GIST)	62	58	100.0	0.0	0.0	0.0
	Appendix, neuroendocrine tumor (NET)	25	14	0.0	0.0	0.0	100.0
	Colorectal, neuroendocrine tumor (NET)	12	9	0.0	0.0	0.0	100.0
	Ileum, neuroendocrine tumor (NET)	53	45	0.0	0.0	0.0	100.0
	Pancreas, neuroendocrine tumor (NET)	101	76	2.6	0.0	3.9	93.4
	Colorectal, neuroendocrine carcinoma (NEC)	14	12	8.3	8.3	8.3	75.0
	Ileum, neuroendocrine carcinoma (NEC)	8	7	0.0	0.0	0.0	100.0
	Gallbladder, neuroendocrine carcinoma (NEC)	4	4	0.0	0.0	75.0	25.0
	Pancreas, neuroendocrine carcinoma (NEC)	14	9	0.0	22.2	11.1	66.7
**Tumors of the urinary system**	Non-invasive papillary urothelial carcinoma, pTa G2 low grade	87	79	77.2	16.5	5.1	1.3
	Non-invasive papillary urothelial carcinoma, pTa G2 high grade	80	73	63.0	26.0	8.2	2.7
	Non-invasive papillary urothelial carcinoma, pTa G3	126	116	36.2	35.3	6.9	21.6
	Urothelial carcinoma, pT2-4 G3	735	520	46.0	25.2	11.9	16.9
	Squamous cell carcinoma of the bladder	22	21	100.0	0.0	0.0	0.0
	Small cell neuroendocrine carcinoma of the bladder	5	5	0.0	20.0	0.0	80.0
	Sarcomatoid urothelial carcinoma	25	12	100.0	0.0	0.0	0.0
	Urothelial carcinoma of the kidney pelvis	62	55	65.5	25.5	1.8	7.3
	Clear cell renal cell carcinoma	1287	1045	10.1	25.6	16.6	47.7
	Papillary renal cell carcinoma	368	275	2.9	5.1	8.7	83.3
	Clear cell (tubulo) papillary renal cell carcinoma	26	18	5.6	5.6	11.1	77.8
	Chromophobe renal cell carcinoma	170	133	30.1	43.6	12.8	13.5
	Oncocytoma of the kidney	257	191	22.0	61.3	13.6	3.1
**Tumors of the male genital organs**	Adenocarcinoma of the prostate, Gleason 3 + 3	83	83	0.0	1.2	2.4	96.4
	Adenocarcinoma of the prostate, Gleason 4 + 4	80	80	0.0	0.0	1.3	98.8
	Adenocarcinoma of the prostate, Gleason 5 + 5	85	85	0.0	0.0	1.2	98.8
	Adenocarcinoma of the prostate (recurrence)	258	237	0.0	1.3	0.8	97.9
	Small cell neuroendocrine carcinoma of the prostate	2	2	0.0	0.0	0.0	100.0
	Seminoma	682	640	95.5	3.9	0.6	0.0
	Embryonal carcinoma of the testis	54	41	82.9	14.6	2.4	0.0
	Leydig cell tumor of the testis	31	30	96.7	0.0	3.3	0.0
	Sertoli cell tumor of the testis	2	2	100.0	0.0	0.0	0.0
	Spermatocytic tumor of the testis	1	1	100.0	0.0	0.0	0.0
	Yolk sac tumor	53	40	60.0	37.5	2.5	0.0
	Teratoma	53	40	72.5	10.0	7.5	10.0
	Squamous cell carcinoma of the penis	92	67	95.5	3.0	1.5	0.0
**Tumors of endocrine organs**	Adenoma of the thyroid gland	63	63	0.0	4.8	12.7	82.5
	Papillary thyroid carcinoma	341	329	0.0	4.6	6.7	88.8
	Follicular thyroid carcinoma	109	106	0.0	7.5	8.5	84.0
	Medullary thyroid carcinoma	57	57	3.5	0.0	1.8	94.7
	Parathyroid gland adenoma	43	41	9.8	31.7	19.5	39.0
	Anaplastic thyroid carcinoma	19	19	84.2	5.3	0.0	10.5
	Adrenal cortical adenoma	48	43	95.3	2.3	2.3	0.0
	Adrenal cortical carcinoma	27	23	60.9	17.4	4.3	17.4
	Pheochromocytoma	51	48	97.9	2.1	0.0	0.0
**Tumors of hematopoetic and lymphoid tissues**	Hodgkin's lymphoma	103	91	100.0	0.0	0.0	0.0
	Small lymphocytic lymphoma, B-cell type (B-SLL/B-CLL)	50	39	100.0	0.0	0.0	0.0
	Diffuse large B cell lymphoma (DLBCL)	113	101	100.0	0.0	0.0	0.0
	Follicular lymphoma	88	77	100.0	0.0	0.0	0.0
	T-cell non-Hodgkin's lymphoma	25	19	100.0	0.0	0.0	0.0
	Mantle cell lymphoma	18	15	100.0	0.0	0.0	0.0
	Marginal zone lymphoma	16	12	100.0	0.0	0.0	0.0
	Diffuse large B-cell lymphoma (DLBCL) in the testis	16	15	100.0	0.0	0.0	0.0
	Burkitt lymphoma	5	2	100.0	0.0	0.0	0.0
**Tumors of soft tissue and bone**	Granular cell tumor	23	13	100.0	0.0	0.0	0.0
	Leiomyoma	50	49	100.0	0.0	0.0	0.0
	Leiomyosarcoma	94	87	100.0	0.0	0.0	0.0
	Liposarcoma	96	82	100.0	0.0	0.0	0.0
	Malignant peripheral nerve sheath tumor (MPNST)	15	13	100.0	0.0	0.0	0.0
	Myofibrosarcoma	26	25	100.0	0.0	0.0	0.0
	Angiosarcoma	42	28	100.0	0.0	0.0	0.0
	Angiomyolipoma	91	69	100.0	0.0	0.0	0.0
	Dermatofibrosarcoma protuberans	21	11	100.0	0.0	0.0	0.0
	Ganglioneuroma	14	11	100.0	0.0	0.0	0.0
	Kaposi sarcoma	8	2	100.0	0.0	0.0	0.0
	Neurofibroma	117	84	100.0	0.0	0.0	0.0
	Sarcoma, not otherwise specified (NOS)	74	63	98.4	0.0	1.6	0.0
	Paraganglioma	41	27	100.0	0.0	0.0	0.0
	Ewing sarcoma	23	12	100.0	0.0	0.0	0.0
	Rhabdomyosarcoma	7	6	83.3	16.7	0.0	0.0
	Schwannoma	122	93	100.0	0.0	0.0	0.0
	Synovial sarcoma	12	9	100.0	0.0	0.0	0.0
	Osteosarcoma	19	10	100.0	0.0	0.0	0.0
	Chondrosarcoma	15	8	100.0	0.0	0.0	0.0
	Rhabdoid tumor	5	5	60.0	0.0	20.0	20.0
	Solitary fibrous tumor	17	17	100.0	0.0	0.0	0.0

**Fig. 3:**
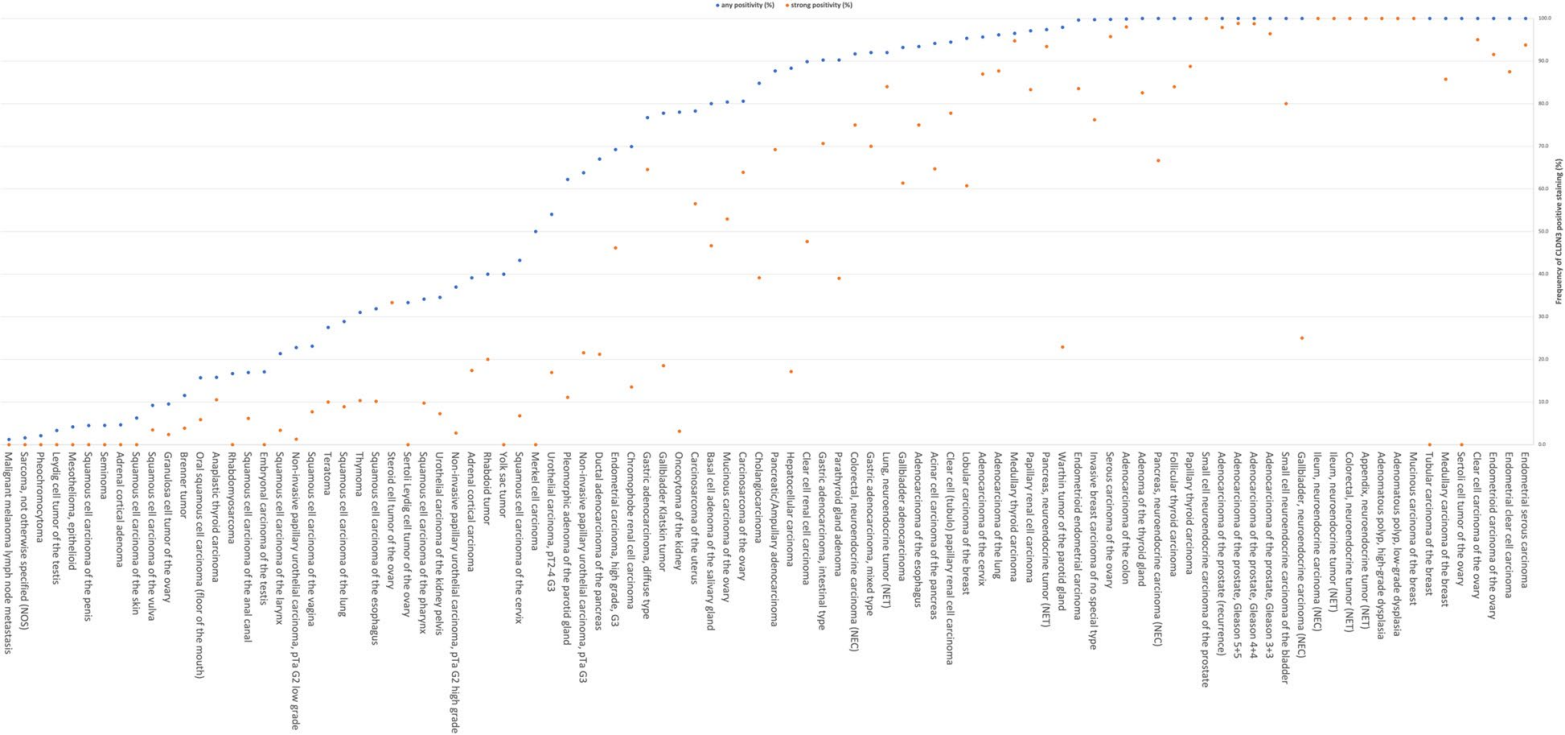
Ranking order of CLDN3 positive immunostaining in different human tumors.In ccRCC, low CLDN3 staining was strongly linked to poor ISUP (*p* < 0.0001), Fuhrman (*p* < 0.0001), and Thoenes (*p* < 0.0001) grades, advanced pT stage (*p* < 0.0001), high UICC stage (*p* = 0.0006), distant metastasis (*p* = 0.0011), as well as shortened overall (*p* = 0.0118; Fig. 4a) and recurrence-free (*p* < 0.0001; Fig. 4b) survival

CLDN3 positivity was most seen in adenocarcinomas (67–100%) and neuroendocrine neoplasms (92–100%) from various organs as well as in other tumors of the female genital tract such as in various subtypes of ovarian and endometrial carcinoma (up to 100%) and different subtypes of breast cancer (95.3–100%). CLDN3 was less common in squamous cell carcinomas (0–43.2%) and mainly absent in melanoma, mesenchymal neoplasia, and in tumors of hematopoetic and lymphoid tissues. A graphical representation of a ranking order of CLDN3 positive and strongly positive cancers is given in Fig. 3.

**Fig. 4:**
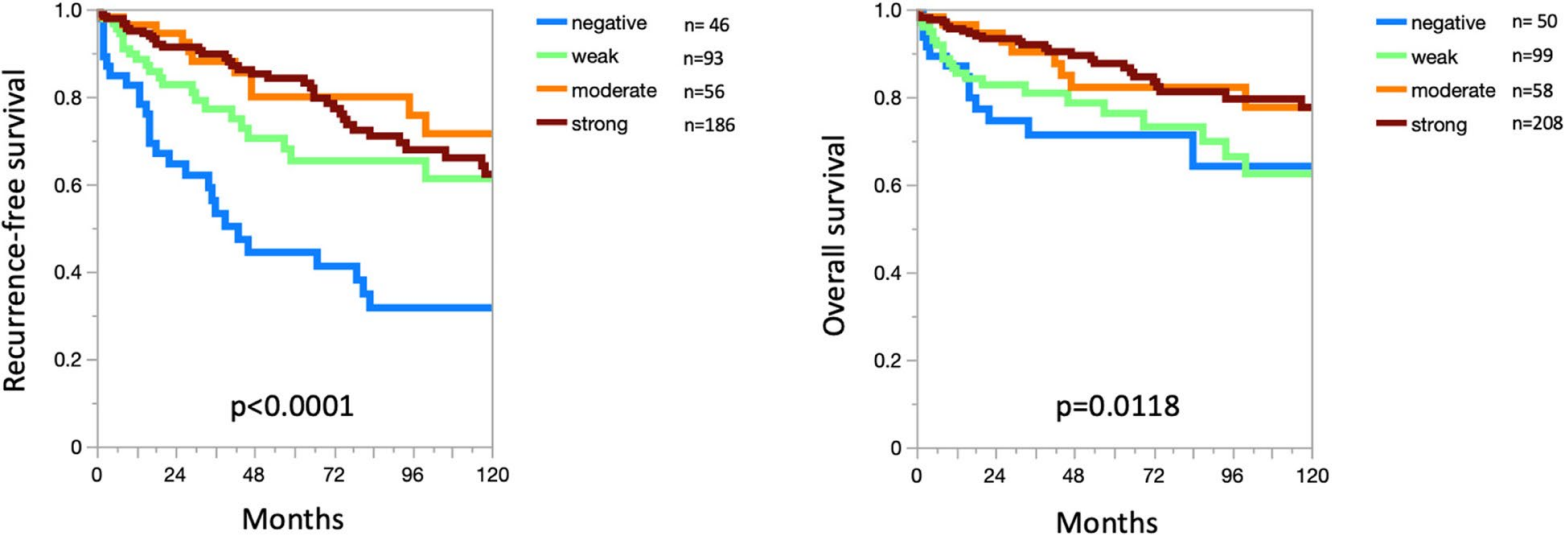
CLDN3 immunostaining and recurrence-free (**A**) and overall survival (**B**) in clear cell renal cell carcinoma

In pRCC, low CLDN3 staining was associated with poor ISUP (*p* = 0.0019), Fuhrman (*p* = 0.0064), and Thoenes (*p* = 0.0315) grades, high pT (*p* = 0.0273), and distant metastasis (*p* = 0.0357). In urothelial carcinoma high CLDN3 staining was associated with high grade in non-invasive carcinomas (*p* < 0.0001), tumor invasiveness (pTa vs. pT2-4; *p* < 0.0001) as well as with nodal metastasis (*p* = 0.0111) and lymphovascular invasion (L1 status; *p* = 0.0062) in the subset of muscle-invasive carcinomas. The level of CLDN3 immunostaining was unrelated to parameters of tumor aggressiveness in ductal adenocarcinoma of the pancreas, gastric cancer and breast cancer. Associations with tumor phenotype are summarized in Table 2.

**Table 2:** CLDN3 immunostaining and tumor phenotype

		**CLDN3 immunostaining**					
		**n**	**negative (%)**	**weak (%)**	**moderate (%)**	**strong (%)**	***p***
**Invasive breast cancer of no special type**	**All cancers**	294	0.3	13.3	9.9	76.5	
	**pT1**	115	0.0	10.4	9.6	80.0	0.1192
	**pT2**	147	0.7	11.6	11.6	76.2	
	**pT3-4**	29	0.0	31.0	3.4	65.5	
	**G1**	8	0.0	0.0	12.5	87.5	0.6289
	**G2**	160	0.0	13.1	10.6	76.3	
	**G3**	126	0.8	14.3	8.7	76.2	
	**pN0**	148	0.7	12.8	9.5	77.0	0.6641
	**pN + **	117	0.0	12.8	12.0	75.2	
**Endometrioid endometrial carcinoma**	**pT1**	105	0.0	6.7	4.8	88.6	0.2436
	**pT2**	23	0.0	8.7	17.4	73.9	
	**pT3-4**	37	0.0	8.1	13.5	78.4	
	**pN0**	50	0.0	4.0	14.0	82.0	0.7811
	**pN + **	29	0.0	6.9	10.3	82.8	
**Clear cell renal cell carcinoma**	**all cancers**	999	10.0	25.9	16.5	47.5	
	**ISUP**						
	**1**	229	5.2	18.3	13.1	63.3	< 0.0001
	**2**	355	5.9	25.4	16.9	51.8	
	**3**	221	10.4	34.4	17.6	37.6	
	**4**	64	48.4	23.4	14.1	14.1	
	**Fuhrman**						
	**1**	54	1.9	11.1	5.6	81.5	< 0.0001
	**2**	598	5.7	24.4	17.4	52.5	
	**3**	252	11.9	32.1	18.7	37.3	
	**4**	78	43.6	28.2	11.5	16.7	
	**Thoenes**						
	**1**	300	6.0	19.7	15.3	59.0	< 0.0001
	**2**	418	9.6	30.4	19.6	40.0	
	**3**	90	31.1	33.3	7.8	27.8	
	**UICC**						
	**1**	261	6.9	24.9	14.6	53.6	0.0006
	**2**	31	12.9	32.3	9.7	45.2	
	**3**	83	21.7	26.5	10.8	41.0	
	**4**	65	26.2	32.3	7.7	33.8	
	**pT1**	574	5.1	23.7	17.1	54.2	< 0.0001
	**pT2**	111	6.3	22.5	20.7	50.5	
	**pT3-4**	303	20.8	31.0	14.2	34.0	
	**pN0**	151	15.2	27.2	11.9	45.7	0.1249
	**pN + **	22	27.3	27.3	22.7	22.7	
	**pM0**	88	9.1	19.3	12.5	59.1	0.0011
	**pM + **	83	25.3	31.3	10.8	32.5	
**Papillary renal cell carcinoma**	**All cancers**	239	2.9	5.9	9.2	82.0	
	**ISUP**						
	**1**	29	0.0	3.4	13.8	82.8	0.0019
	**2**	113	0.9	4.4	3.5	91.2	
	**3**	62	6.5	8.1	17.7	67.7	
	**4**	4	25.0	25.0	25.0	25.0	
	**Fuhrman**						
	**1**	2	0.0	0.0	0.0	100	0.0064
	**2**	153	0.7	3.9	5.9	89.5	
	**3**	63	6.3	7.9	15.9	69.8	
	**4**	8	12.5	12.5	37.5	37.5	
	**Thoenes**						
	**1**	45	0.0	4.4	13.3	82.2	0.0315
	**2**	128	2.3	6.3	7.0	84.4	
	**3**	14	14.3	0.0	28.6	57.1	
	**UICC**						
	**1**	77	1.3	3.9	9.1	85.7	0.0625
	**2**	9	0.0	22.2	22.2	55.6	
	**3**	3	0.0	0.0	0.0	100.0	
	**4**	9	22.2	22.2	11.1	44.4	
	**pT1**	169	1.8	5.3	5.3	87.6	0.0273
	**pT2**	39	2.6	7.7	17.9	71.8	
	**pT3-4**	25	12.0	8.0	16.0	64.0	
	**pN0**	20	0.0	10.0	15.0	75.0	0.2338
	**pN + **	12	16.7	8.3	16.7	58.3	
	**pM0**	23	0.0	4.3	17.4	78.3	0.0357
	**pM + **	11	18.2	18.2	27.3	36.4	
**Ductal adenocarcinoma of the pancreas**	**pT1**	8	50	25	0	25	0.662
	**pT2**	43	37.2	39.5	9.3	14	
	**pT3**	242	35.5	33.9	12.8	17.8	
	**pT4**	17	52.9	29.4	11.8	5.9	
	**G1**	10	50	20	0	30	0.092
	**G2**	218	35.3	33	11.9	19.7	
	**G3**	68	41.2	38.2	13.2	7.4	
	**pN0**	65	40	29.2	12.3	18.5	0.8041
	**pN + **	244	36.1	35.7	11.9	16.4	
**Gastric adenocarcinoma**	**All cancers**	316	13.9	13.9	9.8	62.7	
	**pT1-2**	42	7.1	7.1	4.8	81.0	0.2596
	**pT3**	103	15.5	11.7	12.6	60.2	
	**pT4**	105	15.2	14.3	10.5	60.0	
	**pN0**	61	13.1	8.2	8.2	70.5	0.5204
	**pN + **	189	13.8	13.8	11.1	61.4	
**Urothelial carcinoma**	**All cancers**	619	50.7	27.6	10.3	11.3	
	**pTa G2 low**	79	77.2	16.5	5.1	1.3	< 0.0001
	**pTa G2 high**	73	63.0	26.0	8.2	2.7	
	**pTa G3**	86	36.0	37.2	9.3	17.4	
	**pT2**	97	50.5	20.6	14.4	14.4	0.5546
	**pT3**	183	46.4	31.1	10.4	12.0	
	**pT4**	92	43.5	28.3	12.0	16.3	
	**G2**	18	55.6	27.8	11.1	5.6	0.6878
	**G3**	354	46.3	27.7	11.9	14.1	
	**pN0**	203	51.2	27.1	12.3	9.4	0.0111
	**pN + **	143	38.5	29.4	11.2	21.0	
	**L0**	146	52.7	27.4	11.0	8.9	0.0062
	**L1**	132	36.4	30.3	11.4	22.0	

*Abbreviations:* G grade, *pM* pathologic status of distant metastasis, *pN* pathologic lymph node status, *pT* pathologic tumor stage, *L* Lymphatic invasion status, *ISUP* International Society of Urologic Pathologists, *UICC* Union for International Cancer Control

## Discussion

**Fig. 5:**
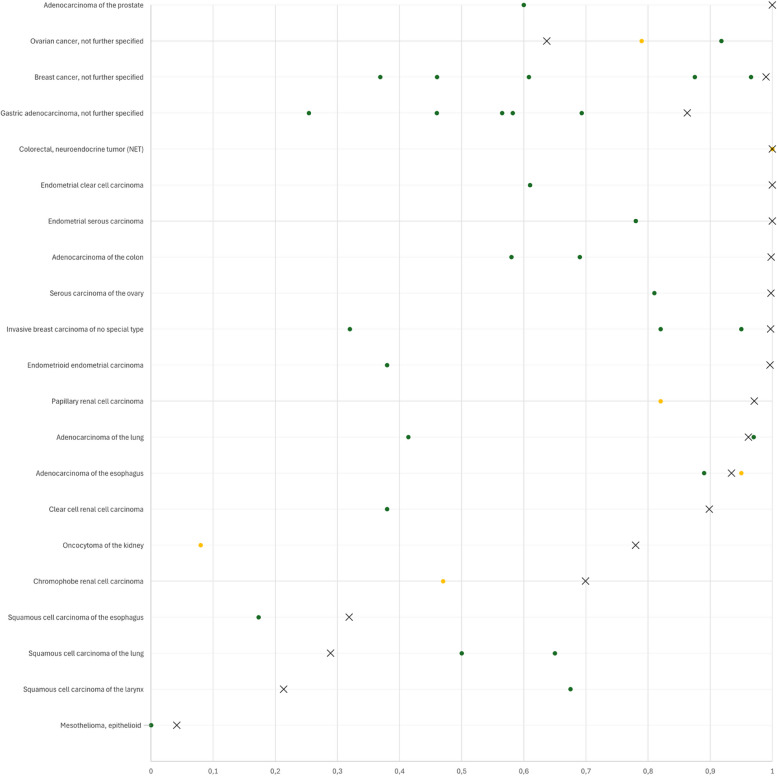
CLDN3 protein expression in cancer (own findings vs. literature data). Graphical representation of CLDN3 data from this study (X) compared to the previous literature. The colors of the dots represent the number of tumors analyzed in these studies: red: *n* ≤ 20; yellow: *n* = 21 to 100; green: *n* > 100. For raw data and references, see suppl. Tab. 1

The results of our successful analysis of 14,966 tumors from 133 different tumor categories provide a comprehensive overview of CLDN3 expression in cancer. Although CLDN3 expression could be found in a wide range of tumor entities, it showed that CLDN3 positivity was most seen in neuroendocrine neoplasms and adenocarcinomas, as well as in tumors of the female genital tract and various subtypes of breast cancer. CLDN3 positivity was less frequent in squamous cell carcinomas and, as described by others [36], only rarely seen in hematolymphoid and in most mesenchymal neoplasms. Although previous studies on CLDN3 expression in cancer were limited in number and had provided partially conflicting data (summarized in Fig. 5), several earlier results are in agreement with our data. For example, CLDN3 positivity was described in 95% of 20 [37] and in 89% of 57 [38] esophageal adenocarcinomas (our study: 93.4%), 100% of 16 colorectal neuroendocrine tumors [39] (our study: 100%), and in 97% of 34 pulmonary adenocarcinomas [29] (our study: 96.2%).

Claudins, which are essential for the formation of TJs in human epithelial and endothelial cells, are altered in a variety of tumors. Because both downregulation and upregulation of CLDN3 have been found in different tumor entities and both alterations have been associated with aggressive tumor characteristics, a tissue type dependency of CLDN3 function has been assumed [40]. The striking associations between a reduced CLDN3 expression and unfavorable histopathological tumor parameters and poor prognosis in ccRCC represents a key finding of our study. As adjuvant systemic therapies are increasingly being administered in high and intermediate risk RCC, there is a need for a better assessment of the individual risk of progression in these tumors [41, 42]. In the future, CLDN3 IHC could evolve towards a clinically useful prognostic marker in RCC, optimally in combination with other markers. A link between reduced CLDN3 expression and poor patient outcome or unfavorable tumor characteristics was previously also found in other cancer types. Jung et al. [21] described an association between reduced CLDN3 expression and L1 status as well as advanced T-stage in a study on 72 gastric adenocarcinomas. Che et al. [24] reported low CLDN3 expression in squamous cell carcinomas of the lung with high pT stage, nodal metastasis, and disease recurrence. Orea et al. [19] found lower disease-free survival and time to clinical progression in prostatic adenocarcinomas with low CLDN3 expression. Jiang et al. [23] found a shortened overall survival in hepatocellular carcinomas with reduced CLDN3 mRNA expression. Functional studies on cell line models identified associations between reduced CLDN3 expression and various cancer driving mechanisms such as a decrease in epithelial barrier function [43], invasiveness [43], dedifferentiation [43], proliferative potential [44], and reduced adhesion [40]. Alternatively, it cannot be excluded that reduced CLDN3 expression in tumors derived from CLDN3 expressing normal cells merely reflects tumor cell dedifferentiation which always parallels cancer progression.

That not only downregulation but also upregulation can be associated with tumor progression in a tumor type dependent manner is demonstrated in our study by the strong relationship between CLDN3 upregulation and grade and stage progression in urothelial carcinomas. This is in line with data from an earlier study by Nakanishi et al. showing a link between high CLDN3 expression and advanced stage, high grade and poor overall survival in a cohort of 129 urothelial cancers of the upper urinary tract [45]. A significant association between CLDN3 upregulation and tumor progression had also been reported for breast [25] and ovarian cancer [17]. Mechanisms that were suggested to explain a tumor promoting role of CLDN3 in cancer include a regulatory impact on cancer stemness [46] and increased chemoresistance [46]. In ovarian cancer cell lines, Agarwal et al. [47] found associations between CLDN3 upregulation and increased cell survival, invasion and motility. Again, it cannot be excluded that CLDN3 neo-expression can be caused by random alterations occurring during dedifferentiation in tumors cell derived from CLDN3 non-expressing normal cells.

Claudins represent potential therapeutic cancer drug targets for several cancer types due to their membranous localization [48]. Initial evidence for druggability of CLDN3 came from experiments with Clostridium perfringens enterotoxin (CPE), which causes food poisoning, and selectively binds to the ECL2 motive of CLDN3 [49, 50]. Non-cytotoxic CPE fragments have therefore been interrogated for their therapeutic potential in cancer. They showed anti-tumor efficacy in prostate [50], breast [51], and ovarian cancer cells [52]. Moreover, C-terminal fragment of CPE increased the efficacy of chemotherapy in ovarian cancer [53] and were also successfully used as a carrier to specifically deliver therapeutic drugs to ovarian cancer cells [54]. While CPE is not specific for CLDN3 but also binds to other claudins, specific antibodies have been developed for treating cancer [7, 55, 56]. Human monoclonal antibodies such as KM390755, IgGH6 [57], and h4G3 [7] been developed against the CLDN3 ECL1 and ECL2 domains. These antibodies were shown to induce antibody-dependent cellular cytotoxicity (ADCC) and in case of KM3907 also a complement-dependent cytotoxicity (CDC) [48, 55].

Considering the large scale of our study, our assay was extensively validated by comparing our IHC findings in normal tissues with data obtained by another independent anti-CLDN3 antibody and CLDN3 RNA data derived from three different publicly accessible databases [58–61]. This validation procedure was suggested by the international working group of antibody validation (IWGAV) [62]. To ensure an as broad as possible range of proteins to be tested for cross-reactivity, 76 different normal tissues categories were included in this analysis. Validity of our assay was supported by the detection of strongest claudin-3 immunostaining in tissues with highest RNA expression (intestine, thyroid, pancreas, and the prostate). True CLDN3 expression in tissues and cell types found to be CLDN3 positive by HMV-309 but lacking documented RNA expression (germinal center cells in lymphatic tissues, megakaryocytes in the bone marrow, squamous epithelium positivity in the thymus and the tonsil crypts, gallbladder, urothelium, placenta, epididymis, gastric mucosa, adrenal gland, and the parathyroidal gland) as well as in tissues with only very low CLDN3 RNA levels (endometrium) were confirmed by identical stainings seen by the independent antibody EPR19971 (Suppl. Figure 1). Given that these CLDN3 positive cell types constituted very small subpopulations of the respective organs, we assume that CLDN3 RNA had not been detected due to a massive dilution if RNAs from total organs were analyzed. Overall, these data document a high specificity of our IHC assay for CLDN3 detection.

## Conclusion

Our data demonstrate significant levels of CLDN3 expression in many different tumor entities, and show that both increased and decreased levels of CLDN3 can occur during tumor progression in a cancer type dependent manner. The strong association between low CLDN3 expression and unfavorable prognostic tumor features may suggest a clinically useful role of CLDN3 expression measurement in ccRCC.

## Supplementary Information

Supplementary Material 1.

Supplementary Material 2.

## Data Availability

All data generated or analyzed during this study are included in this published article.

## Abbreviations

CLDN3: Claudin 3
TJ: Tight junction
RCC: Renal cell carcinoma
ccRCC: Clear cell renal cell carcinoma
pRCC: Papillary renal cell carcinoma
IHC: Immunohistochemistry
TMA: Tissue microarray
CPE: Clostridium perfringes enterotoxin
ADCC: Antibody-dependent cellular cytotoxicity
CDC: Complement-dependent cytotoxicity
IWGAV: International working group of antibody validation
G: Grade
pM: Pathologic status of distant metastasis
pN: Pathologic lymph node status
pT: Pathologic tumor stage
L: Lymphatic invasion status
ISUP: International Society of Urologic Pathologists
UICC: Union for International Cancer Control
