# Supplementary material for: Prevalence and clinical significance of Claudin-3 expression in cancer: a tissue microarray study on 14,966 tumor samples
Source: Biomark Res. 2024 Dec 10;12:154. doi: 10.1186/s40364-024-00702-w (PMC11633013; doi:10.1186/s40364-024-00702-w)
Supplement: Supplementary file 1 — Supplementary Material 1. [file 40364_2024_702_MOESM1_ESM.docx]

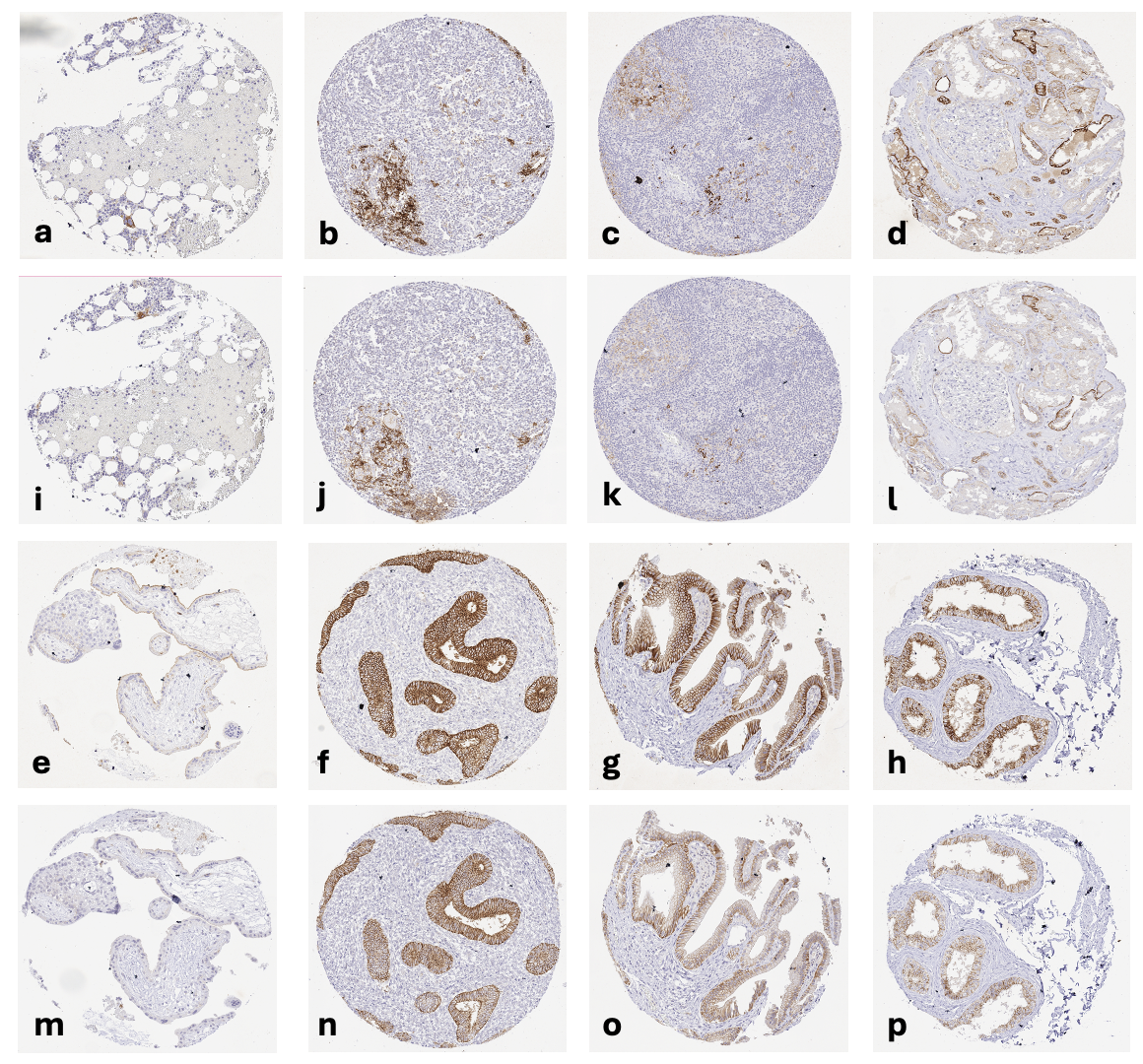


**Suppl. Figure 1.**

**Immunohistochemistry (IHC) validation by comparison of antibodies.** The panels show a concordance of immunostaining results obtained by two independent CLDN3 antibodies. Using HMV-309, a predominantly membranous staining is seen in megacaryocytes of the bone marrow (**a**), a subset of cells of corpuscles of Hassall’s in the thymus (**b**), distinct cell types in a lymph node (**c**), a subset of renal tubular cells (**d**), surface membrane of the syncytiotrophoblast of the early placenta (**e**), endometrium glands (**f**), gallbladder epithelium (**g**), and a subset of epithelial cells in the cauda epididymis (**h**). Using clone EPR19971, a comparable staining was seen in the bone marrow (**i**), thymus (**j**), lymph nodes (**k**), renal tubular cells (**l**), placenta (**m**), endometrium (**n**), gallbladder (**o**), and the epididymis (**p**). The images a-h and i-p are from consecutive tissue sections.
