# Supplementary material for: Prevalence and clinical significance of Claudin-3 expression in cancer: a tissue microarray study on 14,966 tumor samples
Source: Biomark Res. 2024 Dec 10;12:154. doi: 10.1186/s40364-024-00702-w (PMC11633013; doi:10.1186/s40364-024-00702-w)
Supplement: Supplementary file 2 — Supplementary Material 2. [file 40364_2024_702_MOESM2_ESM.docx]

**Suppl Table 1.**

**List of raw data and references used to create Figure 5.**

| **Publication** | **PMID** | **Tumor entity** | **Number of tumors (n)** | **positivity %** |
| --- | --- | --- | --- | --- |
| **Tokuhara et al. 2018** | 29285188 | Adenocarcinoma of the colon | 100 | 58.00% |
| **Ishikawa et al. 2008** | 18181096 | Adenocarcinoma of the colon | 71 | 69.00% |
| **Takala et al. 2007** | 17614851 | Adenocarcinoma of the esophagus | 20 | 95.00% |
| **Montgomery et al. 2006** | 16540726 | Adenocarcinoma of the esophagus | 57 | 89.00% |
| **Chaouche-Mazouni et al. 2015** | 25935651 | Adenocarcinoma of the lung | 34 | 97.00% |
| **Jung et al. 2009** | 19231096 | Adenocarcinoma of the lung | 87 | 41.40% |
| **Szade et al. 2021** | 33147053 | Breast cancer, not further specified | 328 | 46.04% |
| **Jääskeläinen et al. 2018** | 29482498 | Breast cancer, not further specified | 197 | 94.90% |
| **Blanchard et al. 2009** | 19387682 | Breast cancer, not further specified | 299 | 84.00% |
| **Tokes et al. 2005** | 15743508 | Breast cancer, not further specified | 56 | 87.50% |
| **Masili-Oku et al. 2017** | 27810697 | Breast cancer, not further specified | 65 | 60.80% |
| **Choi et al. 2012** | 23018247 | Breast cancer, not further specified | 122 | 36.90% |
| **Inoescu Popescu et al. 2013** | 23529315 | Breast cancer, not further specified | 20 | 75.00% |
| **Ricardo et al. 2012** | 22936447 | Breast cancer, not further specified | 450 | 96,44% |
| **Lechpammer et al. 2008** | 18587324 | Chromophobe renal cell carcinoma | 17 | 47.00% |
| **Lechpammer et al. 2008** | 18587324 | Clear cell renal cell carcinoma | 90 | 38.00% |
| **Ishida et al. 2009** | 19082451 | Colorectal, neuroendocrine tumor (NET) | 16 | 100.00% |
| **Knoecny et al. 2008** | 18313739 | Endometrial clear cell carcinoma | 23 | 61.00% |
| **Knoecny et al. 2008** | 18313739 | Endometrial serous carcinoma | 89 | 78.00% |
| **Knoecny et al. 2008** | 18313739 | Endometrioid endometrial carcinoma | 138 | 38.00% |
| **Jung et al. 2011** | 20462599 | Gastric adenocarcinoma | 72 | 73.60% |
| **Jun et al. 2014** | 24333468 | Gastric adenocarcinoma | 134 | 25.40% |
| **Wang et al. 2015** | 25755790 | Gastric adenocarcinoma | 92 | 56.50% |
| **Zhang et al 2018** | 29749528 | Gastric adenocarcinoma | 122 | 58.20% |
| **Matsuda et al. 2007** | 17459057 | Gastric adenocarcinoma | 94 | 46.00% |
| **Semba et al. 2008** | 18477216 | Gastric adenocarcinoma | 73 | 71% |
| **Skalova et al. 2019** | 31572543 | Invasive breast carcinoma of no special type | 62 | 95% |
| **Skalova et al. 2019** | 31572543 | Invasive breast carcinoma of no special type | 62 | 82% |
| **Lu et al. 2013** | 23222490 | Invasive breast carcinoma of no special type | 226 | 32.00% |
| **Chaouche-Mazouni et al. 2015** | 25935651 | Mesothelioma, epithelioid | 28 | 0% |
| **Kojima et al. 2010** | 20204275 | nasopharyngeal carcinoma | 18 | 55.60% |
| **Lechpammer et al. 2008** | 18587324 | Oncocytoma of the kidney | 12 | 8.00% |
| **Inoescu Popescu et al. 2013** | 23529315 | Ovarian cancer | 19 | 78.95% |
| **Lu et al. 2004** | 15161682 | Ovarian cancer | 158 | 92.00% |
| **Lechpammer et al. 2008** | 18587324 | Papillary renal cell carcinoma | 22 | 82.00% |
| **Choi et al. 2007** | 17647191 | Serous carcinoma of the ovary | 84 | 81.00% |
| **Takala et al. 2007** | 17614851 | Squamous cell carcinoma of the esophagus | 54 | 17.30% |
| **Zhou et al. 2019** | 31115553 | Squamous cell carcinoma of the larynx | 80 | 67.50% |
| **Che et al. 2015** | 25820701 | Squamous cell carcinoma of the lung | 103 | 65.00% |
| **Jung et al. 2009** | 19231096 | Squamous cell carcinoma of the lung | 84 | 50.00% |
| **Nakanishi et al. 2008** | 18550469 | Urothelial carcinoma | 129 | 73.60% |
